# Lichens and mosses as polonium and uranium biomonitors on Sobieszewo Island

**DOI:** 10.1007/s10967-016-5079-8

**Published:** 2016-10-25

**Authors:** Alicja Boryło, Grzegorz Romańczyk, Bogdan Skwarzec

**Affiliations:** Department of Analytical and Environment Radiochemistry, Faculty of Chemistry, University of Gdańsk, Wita Stwosza 63, 80-308 Gdańsk, Poland

**Keywords:** Moss, Lichen, Polonium, Uranium, Activity ratio, Biomonitor

## Abstract

In the study the activities of polonium ^210^Po and uranium ^234^U, ^238^U radionuclides in moss and lichen samples were determined using the alpha spectrometry. Different lichens and mosses were collected around the Sobieszewo Island (northern Poland) and investigated for potential use as biomonitors for ^210^Po and ^238^U deposition. Mosses and lichens have a high efficiency in capturing ^210^Po and ^238^U from atmospheric fallout. The obtained results showed that ^210^Po, ^238^U concentrations are changing in analyzed thallophytes samples depending on the type of thallus.

## Introduction

The lichen is a composite organism that arises from algae or cyanobacteria (or both) living among filaments of a fungus in a symbiotic relationship. The combined life form has properties that are very different from the properties of its component organisms. Lichens come in many colors, sizes, and forms. The properties are sometimes plant-like, but lichens are not plants. Lichens may have tiny, leafless branches (fruticose), flat leaf-like structures (foliose), flakes that lie on the surface like peeling off paint (crustose) or other growth forms [1, 2]. As mentioned above lichens grow in a wide range of shapes and forms. The shape of a lichen is usually determined by the organization of the fungal filaments. The nonreproductive tissues, or vegetative body parts, are called the thallus. Lichens are grouped by thallus type, since the thallus is usually the most visually prominent part of the lichen. Thallus growth forms typically correspond to a few basic internal structure types. Common groupings of lichen thallus growth forms are: fruticose, foliose, leprose, gelatinous, filamentous, byssoid and structureless. Due to the lack of root system, they depend on surface absorption of nutrients and accumulate fallout radionuclides from atmosphere. In these plants, the accumulation degree is much higher than in vascular plants growing in the same habitats [3]. Lichens are well known to accumulate and retain a variety of contaminants, particularly heavy metals and radionuclides [4–6]. Recent literature referring to lichen biomonitoring has dealt mostly with airborne elements emitted by power plants using fossil fuels [5]. Lichens are commonly used in bio-monitoring studies to determine spatial and temporal gradients in air pollution; epiphytic lichens obtain most of their nutrients from the atmosphere in the form of wet and dry deposition of aerosols and gases [7]. Lichens do not contain waxy cuticles or root systems like vascular plants and they can be sensitive to air pollutants, particularly SO_2_ and NO_*x*_, and have served as an indicator of adverse environmental conditions on local, regional and global spatial scales [7]. The obtained results concerning the differences between mosses and lichens as accumulation biomonitors depend on the species and the environment of the researched area. There is rich literature on the use of lichens and mosses as bio-monitors of atmospheric contamination, the organisms are also good radionuclide bioaccumulators (e.g. were used to assess radionuclide fall-out after the Chernobyl accident) [8–19]. Mosses and peat are highly efficient at capturing ^210^Pb and ^210^Po from atmospheric fallout and exhibit high inventory of ^210^Pb and ^210^Po in the order of 0.5–5 kBq m^−2^. The high efficiency by mosses and peat of capturing airborne ^210^Po and ^210^Pb makes them useful as bio-indicators of environmental radioactive contamination [20], In the region of Katirli Mountain in north western Turkey, mosses have been collected as bio-indicators of environmental radioactive contamination of airborne ^210^Po and ^210^Pb activities [21].

The aim of the study was to determinate of ^210^Po, ^234^U and ^238^U concentration and value of the activity ratio between ^234^U and ^238^U as well as to show differences between kind of thallus, individual species and sampling sites taking into account seasonal changes. In order to examine impact of seasons on observed concentration values, partial results from our previous work was included.

## Materials and methods

### Sites sampling

**Fig. 1:**
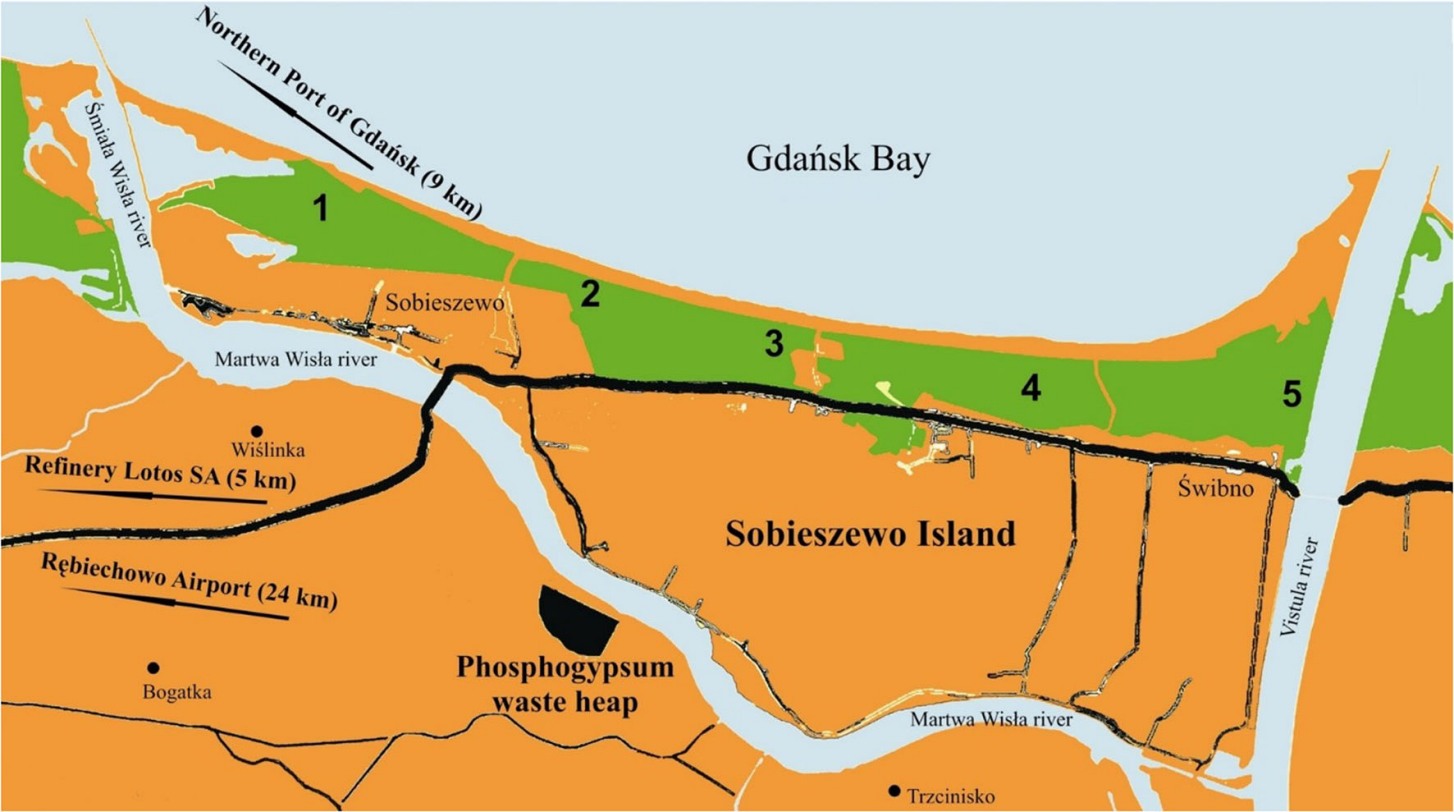
Moss and lichen samples collection sites [22]

The mosses sampled including *Dicranum scoparium* and *Pleurozium schreberi* were collected in spring and autumn 2009 from Sobieszewo Island near the phosphogypsum waste heap in Wiślinka (northern Poland). The lichen samples including *Lepraria incana*, *Lecanora carpinea*, *Evernia prunastri*, *Pseudevernia furfuracea* and *Platismatia glauca* were collected in spring and autumn 2011 from Sobieszewo Island, too. Five positions where mosses and lichens were collected were marked along the area of Sobieszewo Island. The locations of the analyzed moss and lichen samples are shown in Fig. 1. The selected green part of mosses and lichens was dried in the air in a well-ventilated area. Then the material was placed in bags for storage of histopathological materials and the process of drying was continued until the so-called air-dry. The dried material was crushed in agalit mortar, samples were packed and stored until analysis in sealed polyethylene bags [22].

### Analytical method

**Fig. 2:**
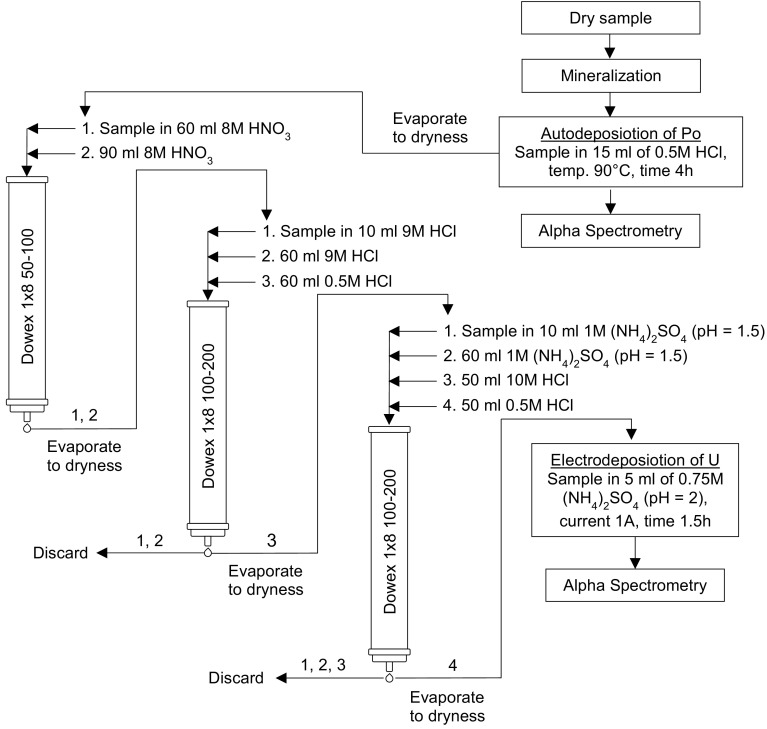
Applied procedure for radiochemical determination ^210^Po, ^234^U and ^238^U

For chemical analysis the green part of mosses, usually representing the last two years of growth were used, while the older brown part of mosses was rejected. Dry moss and lichen samples (about 1–2 g) were mineralized using concentrated acids HNO_3_ and HCl in a volume ratio of 2:1. Following the same procedure for blank samples were analyzed. Before radiochemical analysis, each sample was enriched with about 25 and 50 mBq of ^209^Po and ^232^U as yield tracers, respectively. After evaporation, the dry residue was dissolved in 20 ml of 0.5 M HCl and, after the addition of ascorbic acid to reduce Fe^3+^, the solution was transferred to teflon (PTFE) vessels equipped with a silver sheet bottom. Polonium was autodeposited at 90 °C for 4 h on silver discs. After polonium electrodeposition the sample was evaporated and the dry residue was dissolved in 60 ml of 8 M HNO_3_. The solution was introduced into a column filled with anion exchange resins AG 1–X 8 (50–100 mesh). The column then was washed with 90 ml of 8 M HNO_3_, and the uranium fraction contained also iron, alkaline earth elements were dissolved in 10 ml of 9 M HCl and introduced into a column filled with anion exchange resins AG 1–X 8 (100–200 mesh). The column was washed with 60 ml of 9 M HCl (in order to remove Cs, Sr, Ra, Ni), and next, U, Fe, Co and Cu retained by the resin were washed with 60 ml of 0.5 M HCl. Eluent was evaporated and dissolved in 10 ml of 1 M (NH_4_)_2_SO_4_ (pH 1.5). In order to separate and purify uranium from Fe, Co and Cu, the solution was introduced into a column filled with anion exchange resins AG 1–X 8 (100–200 mesh), and washed with 60 ml of 1 M solution (NH_4_)_2_SO_4_ (pH 1.5) and 50 ml of 10 M HCl subsequently, and next uranium adsorbed by resins was eluted with 50 ml of 0.5 M HCl. The solution containing uranium was evaporated and dry residue was mineralized with 2 ml of 1:1 mixture of concentrated HNO_3_ and HCl. After evaporation, residue was dissolved in 5 ml of 0.75 M (NH_4_)_2_SO_4_ (pH 2) and transferred into cell and the electrolysis was carried out on steel disc during 90 min at a constant current of 1.0 A [22–25]. The activities of ^210^Po, ^234^U, and ^238^U in moss and lichen samples were measured using an alpha spectrometer (Alpha Analyst S470) equipped with semiconductor silicon detectors and 300 mm^2^ active surface barrier (Canberra-Packard, USA). Minimum detectable activity (MDA) was calculated as 0.1 mBq for ^210^Po and 0.3 mBq for ^238^U. Polonium samples were measured for 3 days and ^210^Po activity was calculated on the time of electrodeposition on silver discs. Uranium samples were measured for 2–7 days. The accuracy and precision of the radiochemical methods were within 10 % based on an international laboratory comparison using International Atomic Energy Agency reference materials (IAEA-384, IAEA-385, IAEA-414). The polonium and uranium recoveries in analyzed samples ranged between 70 and 95 %. The results of ^210^Po, ^234^U, and ^238^U concentrations in analyzed samples are given with standard deviation (SD) calculated for a 95 % confidence interval (±2 σ). The obtained results of polonium and uranium radionuclide concentration in analyzed moss and lichen samples are given as an average of four experiments conducted for each sample. The methods of polonium and uranium in mosses samples were perfectly described in previously article titled “Polonium (^210^Po), uranium (^234^U, ^238^U) isotopes and trace metals in mosses from Sobieszewo Island, northern Poland” [22]. Used method was presented on Fig. 2.

## Results and discussion

### ^210^Po and ^238^U concentration in moss samples

**Fig. 3:**
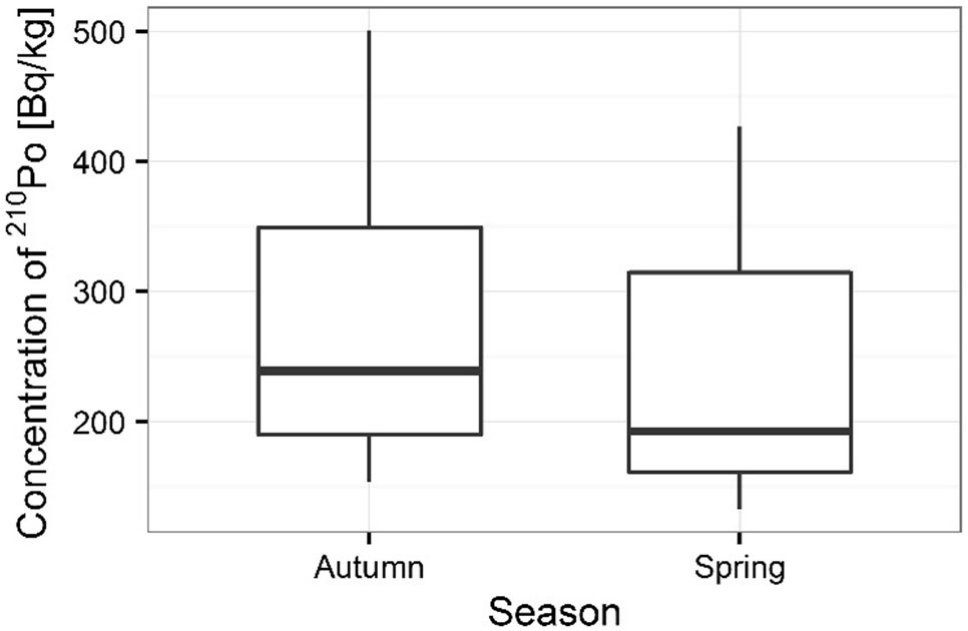
Seasonal concentrations of ^210^Po in analyzed moss samples

**Fig. 4:**
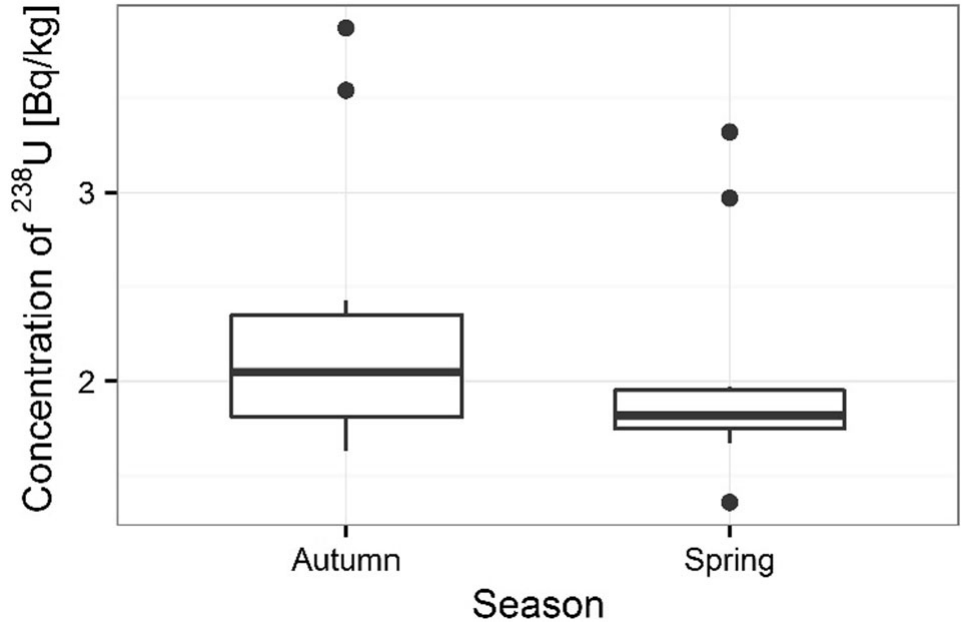
Seasonal concentrations of ^238^U in analyzed moss samples

**Fig. 5:**
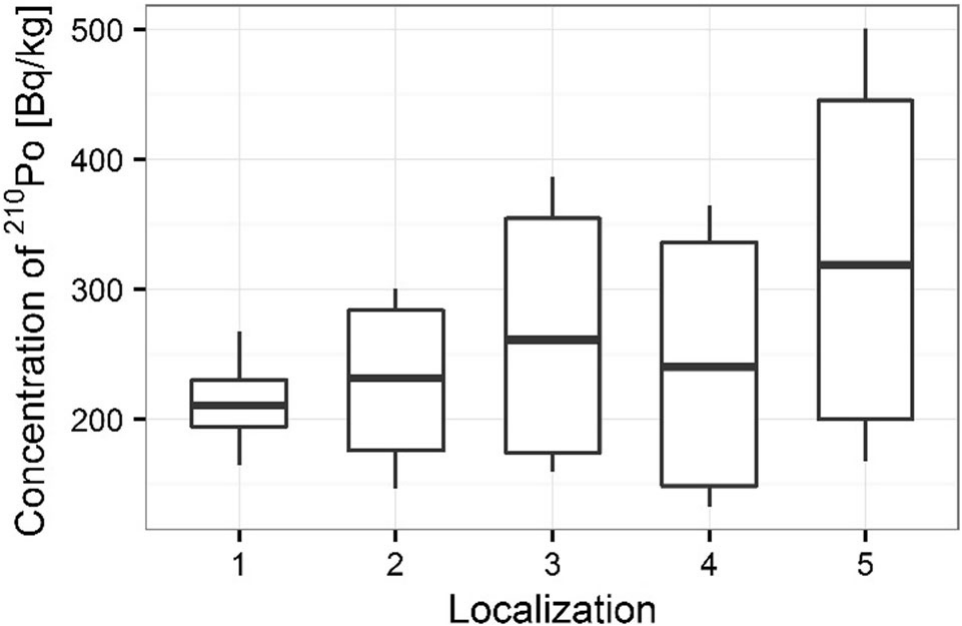
Concentration of ^210^Po for moss samples collecting sites

**Fig. 6:**
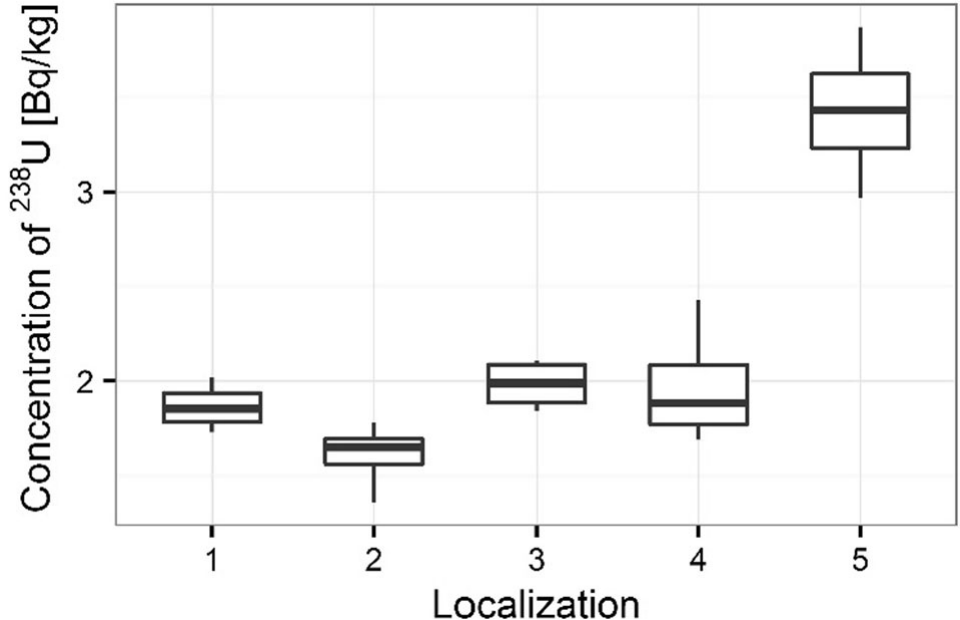
Concentration of ^238^U for moss samples collecting sites

Activity concentrations of ^210^Po and ^238^U radionuclides in moss and lichen samples are listed in Table 1. ^210^Po concentrations in moss samples were between 133 ± 1 and 501 ± 17 Bq kg^−1^, while ^238^U concentration ranged from 1.36 ± 0.13 to 3.87 ± 0.10 Bq kg^−1^. The higher concentrations of ^210^Po and ^238^U were measured in mosses collected in autumn than spring (from 133 ± 1 to 427 ± 15 Bq kg^−1^ in spring [22] and from 154 ± 3 to 501 ± 17 Bq kg^−1^ in autumn for ^210^Po; from 1.36 ± 0.13 to 3.32 ± 0.11 Bq kg^−1^ in spring [22] and from 1.63 ± 0.12 to 3.87 ± 0.10 Bq kg^−1^ in autumn for ^238^U) (Table 1) (Figs. 3, 4). Performed statistical analysis did not confirm the significant differences between the concentrations of ^210^Po and ^238^U in spring and autumn (ANOVA, *p* = 0.380 for ^210^Po and Kruskal–Wallis test, *p* = 0.257 for ^238^U). This effect was described by other research, which shows that the morphology of mosses does not vary with seasons and lichen and moss species retain and accumulate pollutants deposited from the atmosphere throughout the year [3, 26, 27]. The highest ^210^Po and ^238^U concentrations in moss samples were observed for two sampling sites: 3 and 5 (Table 1); (Figs. 5, 6). Statistical analysis of ^210^Po and ^238^U concentrations in moss samples between collection sites shows significant differences only for ^238^U (ANOVA, *p* < 0.001), while no significant differences were found for ^210^Po (ANOVA, *p* = 0.850). The other authors show there were significant differences in concentrations of ^210^Po depending on the place of sampling and the differences observed between the different sampling stations could be connected with various ecological conditions and individual lichen and moss characteristics [3]. The values of the activity ratio between ^234^U and ^238^U isotopes in analyzed moss samples were close to one (from 0.97 ± 0.05 to 1.02 ± 0.08).

**Table 1:** Concentration of polonium ^210^Po, uranium ^238^U, total uranium and values of the activity ratio ^234^U/^238^U in mosses and lichen samples from Sobieszewo Island

Species	Place of sampling	^210^Po concentration (Bq kg^−1^) dry wt		^238^U concentration (Bq kg^−1^) dry wt		Total uranium concentration (mg kg^−1^) dry wt		Activity ratio ^234^U/^238^U	
		Spring	Autumn	Spring	Autumn	Spring	Autumn	Spring	Autumn
*Pleurozium schreberi*	1	218 ± 14^a^	268 ± 15	1.80 ± 0.19^a^	2.02 ± 0.14	0.15 ± 0.04^a^	0.17 ± 0.02	1.00 ± 0.07^a^	1.00 ± 0.07
	2	278 ± 14^a^	301 ± 16	1.67 ± 0.14^a^	1.78 ± 0.15	0.14 ± 0.02^a^	0.16 ± 0.02	0.98 ± 0.07^a^	0.99 ± 0.05
	3	344 ± 11^a^	387 ± 13	1.84 ± 0.23^a^	2.08 ± 0.13	0.16 ± 0.02^a^	0.17 ± 0.02	0.97 ± 0.06^a^	1.02 ± 0.07
	4	327 ± 11^a^	365 ± 12	1.80 ± 0.18^a^	1.69 ± 0.12	0.15 ± 0.04^a^	0.14 ± 0.02	1.00 ± 0.07^a^	0.98 ± 0.06
	5	427 ± 15^a^	501 ± 17	2.97 ± 0.19^a^	3.54 ± 0.11	0.26 ± 0.05^a^	0.29 ± 0.01	0.97 ± 0.03^a^	0.99 ± 0.07
*Dicranum scoparium*	1	165 ± 9^a^	204 ± 10	1.73 ± 0.14^a^	1.91 ± 0.14	0.15 ± 0.04^a^	0.16 ± 0.02	1.00 ± 0.05^a^	0.97 ± 0.05
	2	147 ± 7^a^	186 ± 8	1.36 ± 0.13^a^	1.63 ± 0.12	0.12 ± 0.01^a^	0.13 ± 0.01	1.00 ± 0.05^a^	1.01 ± 0.05
	3	160 ± 9^a^	179 ± 10	1.90 ± 0.14^a^	2.11 ± 0.10	0.16 ± 0.03^a^	0.17 ± 0.01	0.99 ± 0.07^a^	1.00 ± 0.06
	4	133 ± 1^a^	154 ± 3	1.97 ± 0.13^a^	2.43 ± 0.13	0.17 ± 0.02^a^	0.20 ± 0.02	0.98 ± 0.04^a^	1.02 ± 0.08
	5	168 ± 6^a^	211 ± 6	3.32 ± 0.11^a^	3.87 ± 0.10	0.28 ± 0.01^a^	0.32 ± 0.01	0.97 ± 0.03^a^	0.99 ± 0.09
*Lepraria incana*	1	301 ± 10	361 ± 14	0.96 ± 0.10	1.12 ± 0.10	0.09 ± 0.01	0.08 ± 0.01	1.00 ± 0.05	1.00 ± 0.06
	2	346 ± 9	389 ± 15	0.67 ± 0.09	0.83 ± 0.09	0.05 ± 0.01	0.07 ± 0.01	1.01 ± 0.08	0.99 ± 0.07
	3	357 ± 10	402 ± 14	0.82 ± 0.08	1.02 ± 0.08	0.07 ± 0.01	0.08 ± 0.01	0.98 ± 0.07	1.01 ± 0.08
	4	302 ± 7	372 ± 14	0.67 ± 0.11	0.94 ± 0.11	0.06 ± 0.01	0.08 ± 0.01	0.98 ± 0.05	1.02 ± 0.09
	5	374 ± 10	499 ± 10	0.89 ± 0.11	1.20 ± 0.11	0.07 ± 0.01	0.10 ± 0.01	0.97 ± 0.08	0.97 ± 0.06
*Lecanora carpinea*	1	275 ± 9	301 ± 10	0.78 ± 0.09	0.89 ± 0.09	0.07 ± 0.01	0.08 ± 0.01	1.00 ± 0.05	0.99 ± 0.05
	2	267 ± 8	307 ± 11	0.76 ± 0.07	0.87 ± 0.08	0.07 ± 0.01	0.07 ± 0.01	0.99 ± 0.06	0.98 ± 0.07
	3	279 ± 10	328 ± 12	0.83 ± 0.10	0.91 ± 0.10	0.07 ± 0.01	0.08 ± 0.01	0.99 ± 0.06	0.98 ± 0.06
	4	278 ± 11	311 ± 10	0.79 ± 0.09	0.88 ± 0.09	0.07 ± 0.01	0.08 ± 0.01	0.99 ± 0.05	0.98 ± 0.08
	5	289 ± 10	334 ± 9	0.89 ± 0.12	1.10 ± 0.10	0.08 ± 0.01	0.09 ± 0.01	0.97 ± 0.04	0.99 ± 0.04
*Evernia prunastri*	1	203 ± 5	243 ± 8	0.56 ± 0.08	0.87 ± 0.08	0.05 ± 0.01	0.07 ± 0.01	1.00 ± 0.07	1.00 ± 0.07
	2	223 ± 6	257 ± 7	0.51 ± 0.10	0.69 ± 0.10	0.04 ± 0.01	0.06 ± 0.01	1.02 ± 0.09	1.02 ± 0.08
	3	278 ± 4	291 ± 9	0.67 ± 0.10	0.81 ± 0.10	0.06 ± 0.01	0.07 ± 0.01	0.98 ± 0.03	1.02 ± 0.07
	4	191 ± 6	211 ± 6	0.35 ± 0.08	0.45 ± 0.08	0.03 ± 0.01	0.04 ± 0.01	0.98 ± 0.04	0.98 ± 0.04
	5	285 ± 8	300 ± 8	0.82 ± 0.10	0.91 ± 0.10	0.07 ± 0.01	0.07 ± 0.01	0.99 ± 0.03	0.97 ± 0.05
*Pseudevernia furfuracea*	1	120 ± 8	156 ± 8	0.54 ± 0.08	0.71 ± 0.04	0.05 ± 0.01	0.06 ± 0.01	0.99 ± 0.05	0.97 ± 0.05
	2	126 ± 8	161 ± 10	0.56 ± 0.08	0.73 ± 0.03	0.05 ± 0.01	0.06 ± 0.01	0.98 ± 0.06	0.98 ± 0.06
	3	132 ± 7	178 ± 9	0.62 ± 0.07	0.84 ± 0.05	0.05 ± 0.01	0.07 ± 0.01	0.98 ± 0.07	0.99 ± 0.07
	4	134 ± 9	171 ± 10	0.58 ± 0.07	0.71 ± 0.06	0.05 ± 0.01	0.06 ± 0.01	0.97 ± 0.05	0.98 ± 0.07
	5	145 ± 6	189 ± 11	0.65 ± 0.09	0.87 ± 0.04	0.06 ± 0.02	0.08 ± 0.01	0.97 ± 0.05	0.97 ± 0.08
*Platismatia glauca*	1	104 ± 2	123 ± 3	0.65 ± 0.08	0.72 ± 0.08	0.05 ± 0.01	0.06 ± 0.01	1.00 ± 0.05	0.98 ± 0.03
	2	114 ± 3	134 ± 4	0.22 ± 0.06	0.32 ± 0.06	0.02 ± 0.01	0.03 ± 0.01	1.01 ± 0.04	0.99 ± 0.07
	3	127 ± 4	156 ± 4	0.61 ± 0.11	0.72 ± 0.11	0.05 ± 0.01	0.06 ± 0.01	1.01 ± 0.07	0.98 ± 0.06
	4	109 ± 4	141 ± 4	0.33 ± 0.07	0.43 ± 0.07	0.03 ± 0.01	0.04 ± 0.01	1.00 ± 0.05	0.97 ± 0.08
	5	148 ± 4	169 ± 5	0.75 ± 0.10	0.84 ± 0.10	0.06 ± 0.01	0.07 ± 0.01	1.00 ± 0.03	1.03 ± 0.08

^a^The results were published in [22]

**Fig. 7:**
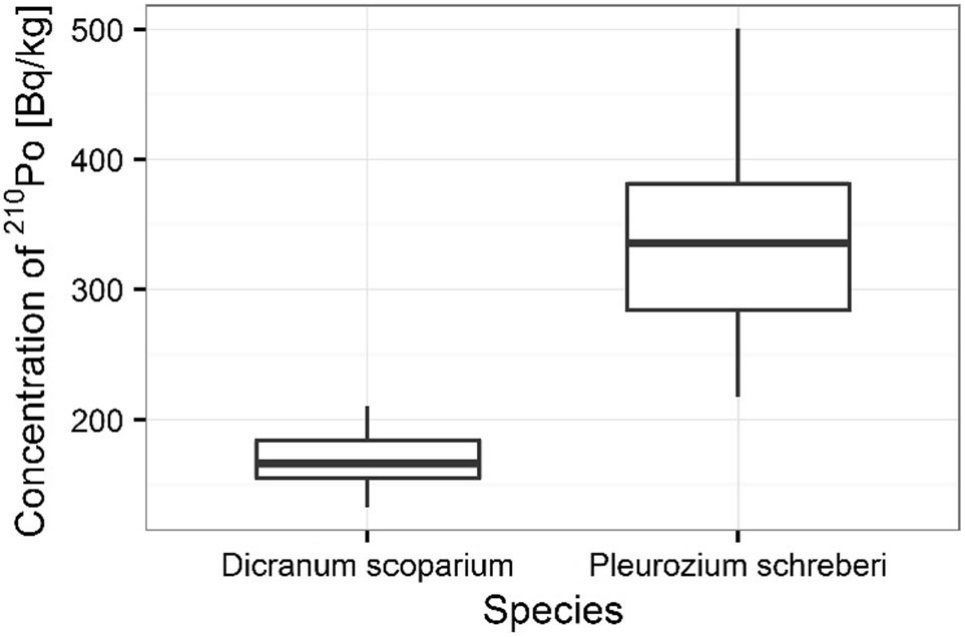
Concentration of ^210^Po in analyzed moss species

**Fig. 8:**
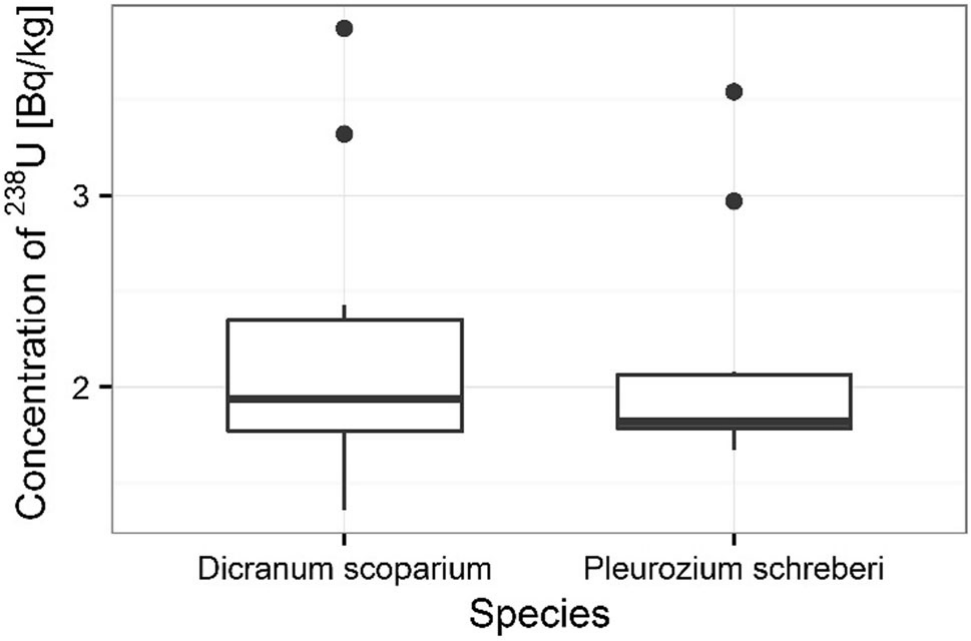
Concentrations of ^238^U in analyzed moss species

The statistical analysis shows significant differences between ^210^Po content in individual mosses species (ANOVA, *p* < 0.001). There were not significant differences between ^238^U content in analyzed mosses species (Kruskal–Wallis, *p* = 0.705). The higher ^210^Po concentration was observed in samples of *P. schreberi*, where its average concentration was 342 ± 26 Bq kg^−1^ respectively, the smaller concentration was measured in *D. scoparium* samples (171 ± 8 Bq kg^−1^) (Fig. 7). The average ^238^U concentration in *P. schreberi* and *D. scoparium* samples was respectively 2.12 ± 0.20 mg kg^−1^ and 2.22 ± 0.25 mg kg^−1^ (Fig. 8).

### ^210^Po and ^238^U concentration in lichen samples

**Fig. 9:**
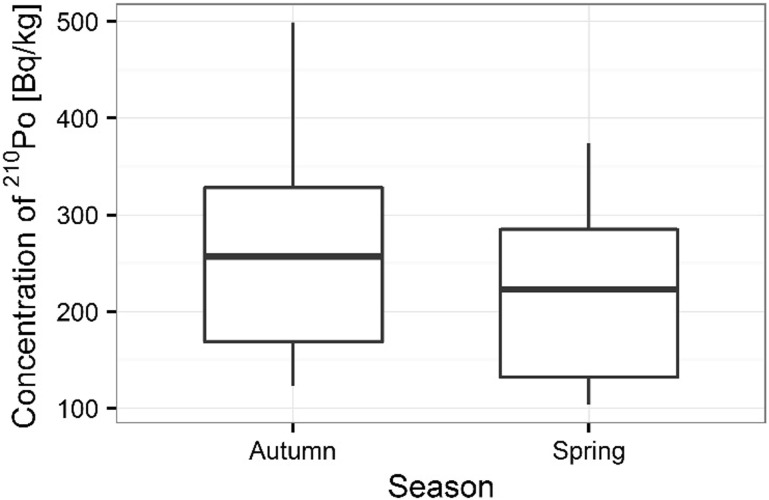
Seasonal concentrations of ^210^Po in analyzed lichen samples

**Fig. 10:**
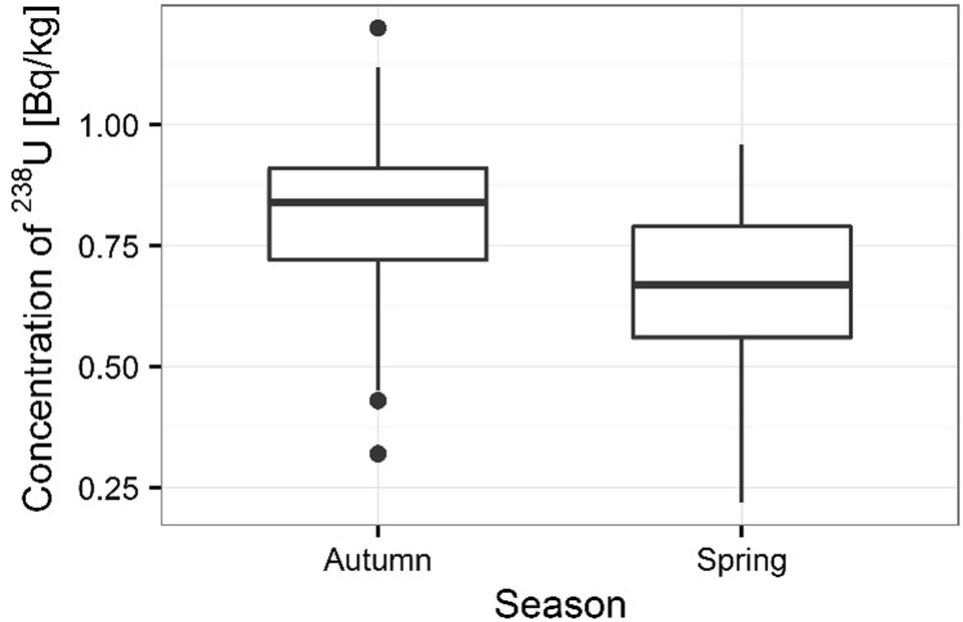
Seasonal concentrations of ^238^U in analyzed lichen samples

^210^Po concentration in lichen samples collected from Sobieszewo Island was in the range between 104 ± 2 to 499 ± 10 Bq kg^−1^, while ^238^U concentration ranged from 0.22 ± 0.06 to 1.12 ± 0.10 Bq kg^−1^ (Table 1). The results of polonium ^210^Po concentrations clearly varied depending on the season (ANOVA, *p* < 0.001). The slightly higher values of ^210^Po concentration were measured, similar as in moss samples, in samples collected in autumn (from 123 ± 3 to 546 ± 9 Bq kg^−1^), smaller in samples collected in spring (from 104 ± 2 to 406 ± 9 Bq kg^−1^) (Fig. 9). ^238^U and total uranium concentration in moss and lichen samples from Sobieszewo Island was varied. The small ^238^U concentration was observed for spring, definitely higher for autumn. The obtained results of the statistical analysis showed significant seasonal differences, too (ANOVA, *p* < 0.001). The similar effect was noticed for ^238^U. Concentration of ^238^U in lichen samples was within a range from 0.22 ± 0.06 to 0.96 ± 0.10 mg kg^−1^ for spring (with the average value 0.66 ± 0.04 mg kg^−1^) and from 0.32 ± 0.07 to 1.20 ± 0.11 mg kg^−1^ (with the average value 0.82 ± 0.04 mg kg^−1^) for autumn (Fig. 10). Performed statistical analysis shows significant differences between the activities of ^238^U in lichen samples and seasons (ANOVA, *p* = 0.004). Thus lichen samples accumulate ^210^Po and ^238^U from the atmosphere throughout the year, wherein the higher ability of accumulation is observed in autumn, while the lower in spring. This effect can be suggest, that the morphology of lichens vary with seasons.

**Fig. 11:**
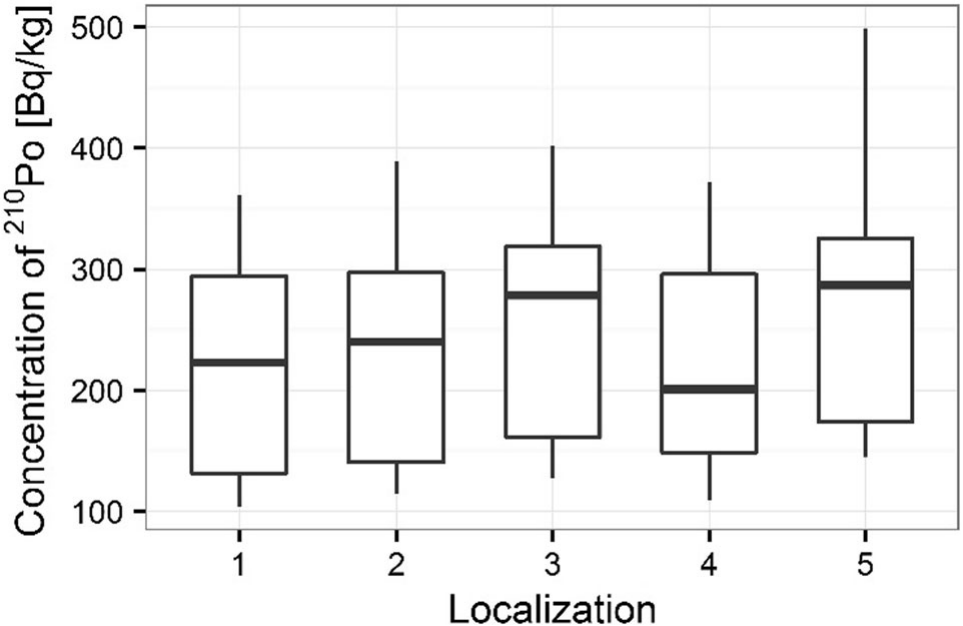
Concentration of ^210^Po for lichen samples collecting sites

**Fig. 12:**
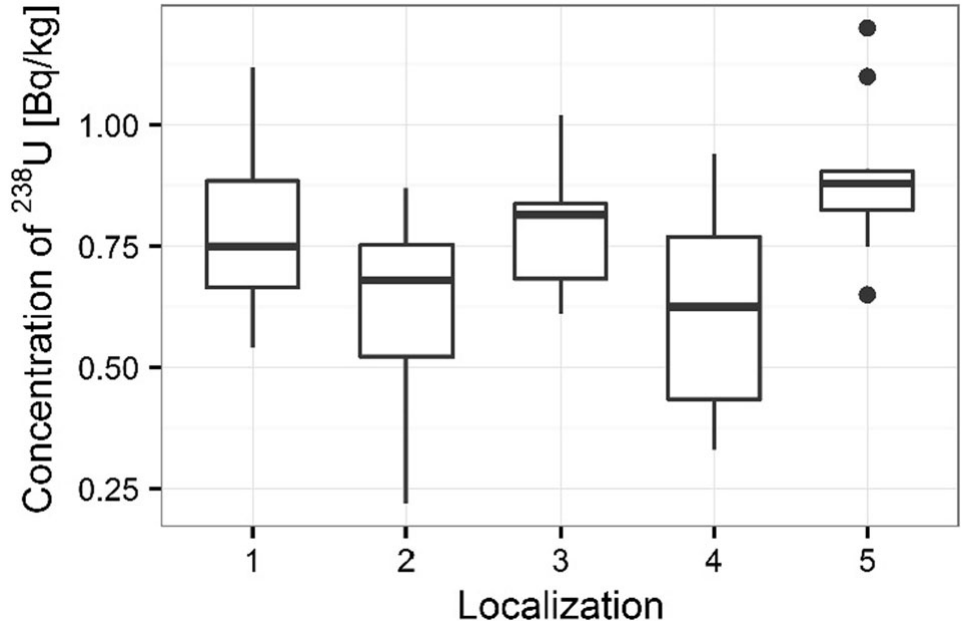
Concentration of ^238^U for lichen samples collecting sites

As in the case of moss samples, the highest ^210^Po and ^238^U concentrations were measured near the positions of samples collection 3 and 5 (Figs. 11, 12). The position number three is situated in the vicinity of Orle settlements belonging to Sobieszewo Island, whereas position number five is situated near Świbno (this place is a branch of the Vistula River in its delta. It is also referred to as *Przekop Wisły*, which can be translated literally from Polish as *Vistula Dug*-*through*). There were significant differences in concentrations of ^238^U in lichen samples among the five areas (ANOVA, *p* = 0.018), while statistically significant differences were not observed while for ^210^Po (ANOVA, *p* = 0.766). The similar effect was observed in moss samples, too. As other studies evidenced the differences observed between the different sampling stations could be connected connection with various ecological conditions, climate type and individual lichen and moss characteristics [3], as well as place of samples collection. Poikolainen [28] indicated that the physiological and morphological properties or behavior of even the same species may change from place to place. This situation can influence the accumulation properties of the same species. The higher values of ^210^Po and ^238^U concentrations in sites 3 and 5 are likely to be related to the location of sampling sites. The sources of analyzed radionuclides in the analyzed species of mosses and lichens in these areas are probably various industrial branches in Gdańsk agglomeration (the petroleum refinery Lotos SA, “Remontowa” Shipyard SA, Gdańsk Power Station, thermal power station in Gdańsk, the “Siarkopol” plant, the lightweight aggregate “Pollytag SA”) [22]. This conclusion can be drawn from the resulting concentration distribution, and, what is important, based on the direction and strength of winds, which were measured in three points (Świbno, Rębiechowo, Port Północny) in the vicinity of Gdansk agglomeration [22]. The perfectly distribution of winds was published in the case of moss samples in spring season from Sobieszewo Island in 2012 [22]. The winds blowing from the southwest could have carried pollutants to site 3, and winds coming from the south could have driven pollutants to site 5 (near phosphogypsum stockpile in Wiślinka). The similar effect was observed by Garty which described the use of lichens as biomonitors around the coal-fired power station in Israel [5]. He shows, that the greater part of winds blowing from various directions drive pollutions to sites located around the Oroth Rabin Power Station near Hadera. Such factors as the wind direction, its velocity and humidity have an impact an radionuclides` content in the environment and can be used to diagnose a long-term atmospheric contamination with polonium and uranium and to identify the contamination source [17]. Some of the biomonitoring studies throughout the world indicated that the concentrations of various elements in lichens and mosses are inversely correlated with the distance from pollution sources [29, 30]. Contrary to other studies in this research, it has been found that relationships between distance from the plant and contamination in mosses and lichens are directly correlated. Our research was carried out at the moment, when phosphogypsum stockpile in Wiślinka was still working. The phosphate rocks contain a lot of natural radionuclides, especially forms of uranium and thorium decay series. The radionuclides of uranium (^234^U, ^235^U, ^238^U), thorium (^232^Th) and existing in the equilibrium with them radionuclides of radium (^226^Ra, ^228^Ra), polonium (^210^Po) and lead (^210^Pb) almost always exist in sedimentary phosphoric rocks. Generally uranium (^234^U, ^238^U), radium (^226^Ra), polonium (^210^Po) and lead (^210^Pb) radionuclides belong to the most radiotoxic, dangerous alpha emitters. Phosphoric acid, the material for the production of phosphate fertilizers is obtained in a wet process by reaction of the phosphatic rocks with sulphuric acid. In this process the uranium is associated with the phosphoric acid fraction, while the ^210^Po and ^210^Pb are bound to the phosphogypsum fraction. In the wet method used by the Gdańsk Phosphate Fertilizer Plant, phosphoric acid is obtained directly as a result of the reaction of phosphate ore with sulphuric acid. In this process the obtained hydrated calcium sulphate is a major component of phosphogypsum (stored in the waste heap). In the next stage of this reaction the obtained solution of phosphoric acid (with pollution) reacts with another portion of phosphate and forms a triple superphosphate. In the wet method about 86 % of the activity of uranium radionuclides and about 70 % of thorium remain in the filtered phosphoric acid, while approximately 85 % of the activity of ^210^Po and ^210^Pb remains with the phosphogypsum. Emission of radionuclides (except radon gas ^222^Rn) to the atmosphere is also very important. The essence of radiotoxicity of phosphogypsum waste heap is not only gamma radioactivity. Natural alpha radioactive elements, which are leached by rains and bioaccumulated in plant and animal organisms as well as in human organism. In longer time they can cause the development of cancer disease. The treatment of phosphoric rocks is the source of natural higher radioactivity, because in the sedimentary phosphoric rocks there are natural radionuclides of uranium (^234^U, ^235^U and ^238^U), thorium (^232^Th), radium (^226^Ra, ^228^Ra), lead (^210^Pb) and polonium (^210^Po). To scale of the potential radiological hazard testifies the fact that in the mid-1990s of the last century there was mined an average of 130 million tons of phosphate rock per year, which included about 150 TBq of ^226^Ra [31]. The produced phosphogypsum contains high activity of ^234^U, ^238^U, ^226^Ra, ^210^Pb and ^210^Po, so its deposition and utilization is important and troublesome for many countries. In some countries the phosphogypsum is removed to sea or oceans. The phosphogypsum waste heap contains most of radiotoxic nuclides: polonium ^210^Po, (and its mother radionuclide ^210^Pb), radium ^226^Ra and uranium (^234^U and ^238^U). In the Wiślinka waste heap the estimated activity in 16 million tons of phosphogypsum contains about 4.51 × 10^11^ Bq for ^234+238^U [22–32].

**Fig. 13:**
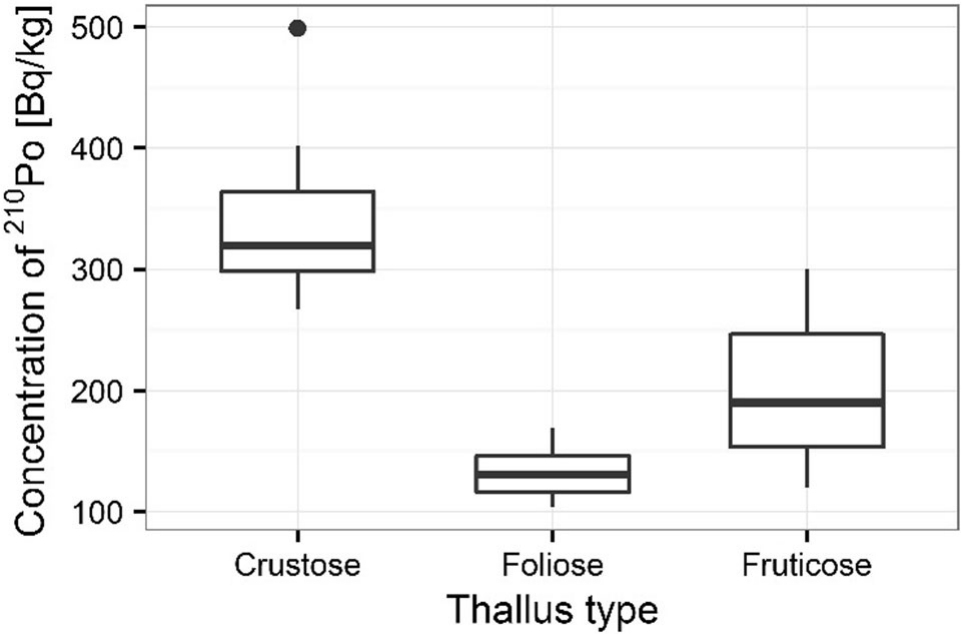
Concentration of ^210^Po in analyzed lichen thallus types

**Fig. 14:**
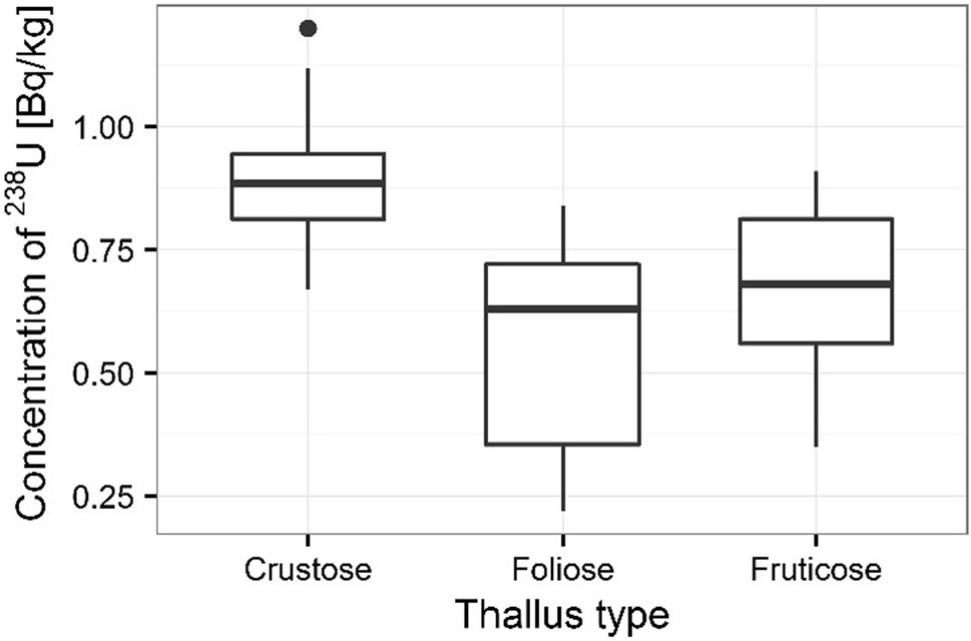
Concentration of ^238^U in analyzed lichen thallus types

Significant differences between bioaccumulation of ^210^Po were measured in individual lichen species given kind of thallus for analyzed organisms (thallus meaning a green shoot ort wig is the undifferentiated vegetative tissue of some organisms in diverse groups such as algae, fungi, some liverworts and lichens). The trapping of relatively large particles on the lichen thalli is a main cause of elevated concentrations of radionuclides and metals in lichens [33]. Irregularities in surfaces of lichens as well as in the shapes of particles may affect attachment to thalli [7]. The highest ^210^Po bioaccumulation capacity was observed in organisms built from crustose thallus (crust-like, adhering tightly to a surface (substrate) like a thick coat of paint) (e.g. *L. incana* and *L. carpinea*), where the average concentration of analyzed radionuclide was 334 ± 13 Bq kg^−1^ (Fig. 13). The smaller ^210^Po bioaccumulation capacity was measured in organisms built from fruticose thallus (growing up like a tuft or multiply branched leafless mini-shrub, or hanging down in strands or tassles) (e.g. *E. prunastri* and *P. furfuracea*), where the average ^210^Po concentration was 200 ± 13 Bq kg^−1^) (Fig. 13). The smallest average ^210^Po concentration (133 ± 7 Bq kg^−1^) was measured in organisms built from foliose thallus (flat, leaf-like lobes that lift up from the surface) (e.g. *P. glauca*) (Fig. 13). The observed differences between ^210^Po bioaccumulation capacity and kind of thallus have also been confirmed statistically using ANOVA (*p* < 0.001). Significant statistical differences based on ANOVA were also found for the characterizing thallus of thallophyte in the case of ^238^U (*p* < 0.001). The highest ^238^U bioaccumulation was observed for lichens with crustose thallus (*L. incana, L. carpinea*) (with the average value 0.89 ± 0.03 mg kg^−1^), slightly smaller for lichens with fruticose thallus (*E. prunastri*, *P. furfuracea*) (with the average value 0.67 ± 0.03 mg kg^−1)^, and the smallest for lichens with foliose thallus (*P. glauca*) (with the average value 0.56 ± 0.07 mg kg^−1^) (Fig. 14). Lichens are very effective in trapping polonium and uranium from the surrounding environment and concentrations of these radionuclides in lichen thalli are directly correlated with the environmental levels of these elements [34]. Additionally, it is known that some species of lichens and mosses are more tolerant of atmospheric pollution [3]. Szczepaniak and Biziuk [27] show that many moss species are geographically widespread and grow in different environmental conditions, even in industrial and urban areas. The kind of lichen thalli and its ability to accumulated heavy metals and radionuclides from the atmosphere is widely discussed in the world [5, 34]. Luigi and others [35] show that the higher uranium concentrations in lichens may be explained by contamination of lichen thalli by soil particles. The filamentous, foliose and fruticose thallus of lichens are especially important [36].

**Fig. 15:**
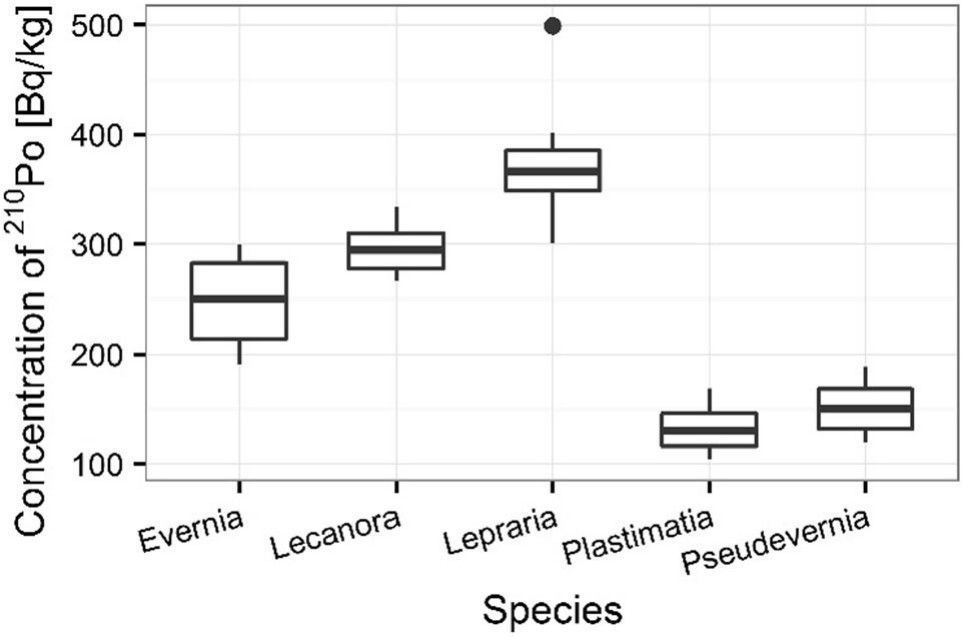
Concentration of ^210^Po in analyzed lichen species

**Fig. 16:**
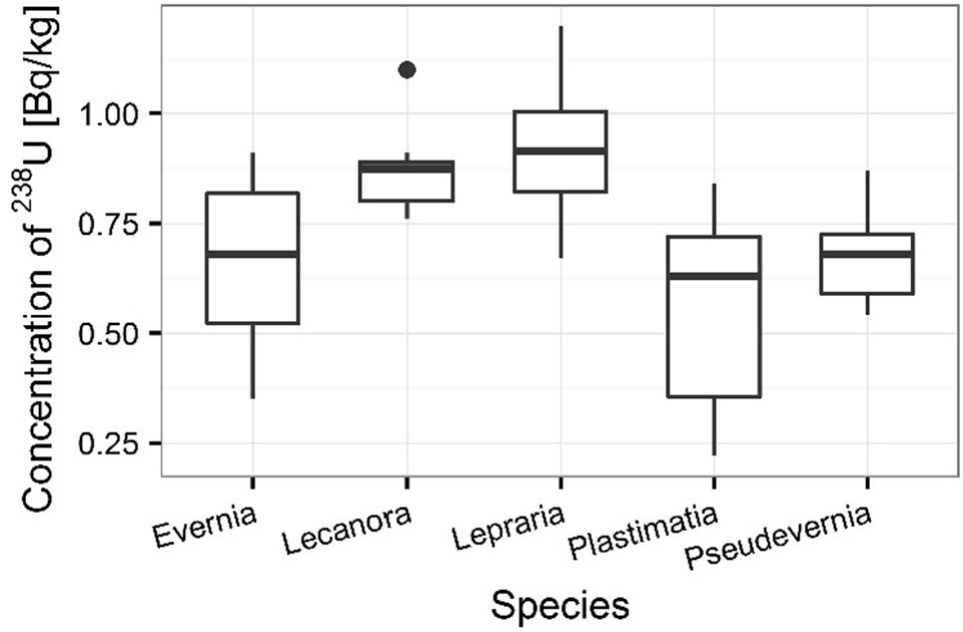
Concentration of ^238^U in analyzed lichen species

Statistically significant difference was also confirmed by ANOVA between the concentration of ^210^Po and ^238^U in different species of lichens (ANOVA, *p* < 0.001 for ^210^Po and ^238^U). The observed changes between individual species are presented in Figs. 15 and 16. The differences observed in ^210^Po and ^238^U concentrations in lichen samples are the results of differences in accumulation properties of species. The other authors show that some lichen and moss species which grow in lower parts of tree trunks are protected from direct radioactive deposition, the low activity concentrations of radionuclides were found in these organisms which collected under trees [3]. The average ^210^Po concentration in lichen samples was 370 ± 18 Bq kg^−1^ for *L. incana*, 297 ± 7 Bq kg^−1^ for *L. carpinea*, 248 ± 13 Bq kg^−1^ for *E. prunastri*, 151 ± 7 Bq kg^−1^ for *P. furfuracea* and 133 ± 7 Bq kg^−1^ *P. glauca* (Fig. 15). The average ^238^U concentration in *L. incana* and *L. carpinea* samples was respectively 0.91 ± 0.06 and 0.87 ± 0.03 mg kg^−1^, in *E. prunastri* and *P. furfuracea* samples 0.66 ± 0.06 and 0.68 ± 0.04 mg kg^−1^ respectively, whereas in *P. glauca* samples it was 0.56 ± 0.07 mg kg^−1^ (Fig. 16). This fact is observed in other research. Sert and others show that the differences observed in ^210^Po concentrations in lichen and moss samples could be linked to the differences in accumulation properties of species. Moreover, the content of polonium in moss and lichen samples depends not only on the kinds individual species but also on the place of sampling. Some lichen and moss species that grow in lower parts of tree trunks are protected from direct radioactive deposition. The different moss and lichen species collected in the same biotope can exhibit differences in their accumulation properties [3].

The values of the activity ratios of ^234^U/^238^U in lichen and moss samples are between 0.97 ± 0.05 and 1.03 ± 0.08, which indicates that the main source of polonium and uranium in the analyzed area is air dry atmospheric fallout which contains phosphogypsum particles from the phosphogypsum waste heap in Wiślinka.

The obtained results of ^210^Po in lichen and moss samples are similar (but significantly lower for lichens) to values of the activity concentrations of ^210^Po collected around coal-fired power plants in Western Turkey (from 151 ± 7 to 593 ± 21 Bq kg^−1^ for mosses and from 124 ± 5 to 1125 ± 38 Bq kg^−1^ for lichens respectively) [3]. The obtained values agreed with the values found in several species in central Sweden, where the level of ^210^Po was 560 ± 70 Bq kg^−1^ with an average activity concentration in lichen 250 Bq kg^−1^, while the higher values were observed in moss samples (from 185 to 960 Bq kg^−1^) [37]. The highest ^210^Po concentrations were defined for environment of Kaiga nuclear power plant site in the south western region of India (2724 ± 13 Bq kg^−1^) [38] and in Gokova region, where Yatagan is located (there are three major coal-fired power plants causing pollution in the surroundings) (from 600 to 1228 Bq kg^−1^) [39] and in the eastern Mediterranean sea region (Syrian coastal mountains series (1322 Bq kg^−1^) [40]. Our values of uranium concentrations are smaller than the values reported for lichens from Balkan area [6] and are perfectly in line with the values reported from Kosovo [35]. The smaller values of ^210^Po concentrations (39–188 Bq kg^−1^) than these obtained in the article were observed in different terrestrial samples collected at Dovrefjell–Sunndalsfjella National Park in Norway in 2007 [41].

Lichens are slowly growing perennials that have high interception potentials for aerosols in precipitation, and therefore contain significantly higher ^210^Po, ^210^Pb and ^238^U concentrations than vascular plants. The measurement of ^210^Po in communities of lichen samples (*Cladonia. Alpestris*) from North Europe indicates average activity concentration of about 250 Bq kg^−1^, and the ^210^Po/^210^Pb activity ratio close to one. The level of ^210^Po and ^210^Pb activity in lichens is quite high and is the consequence of their extensive grazing of lichen [20].

The obtained results indicate that the area of Sobieszewo Island is an area with slight air pollution, because there are crustose lichens, fruticose and foliose lichens. Based on the scale of lichen, by which, through observations of types of fronds of lichen growing on the bark of deciduous trees, you can assess the level of air pollution in the area, we can distinguish here zone V an VI. The zone V is characterized by relatively little air pollution and occurrence of fruticose lichens (e.g. *E. prunastri* and *P. furfuracea*). The zone VI is sensitive to pollution and represented by foliose lichens (e.g. *P. glauca*).

## Conclusions

The polonium and uranium content in lichen and moss samples as well as isotopic ratios ^234^U/^238^U was measured by alfa-spectrometer technique. The results of the presented survey suggest that the lichens and mosses can be good indicators of polonium and uranium contamination in environment. The present results also proved that the higher polonium and uranium content determined for mosses than lichens. ^210^Po concentrations were found higher than ^238^U concentrations at all sampling stations. The highest polonium and uranium concentrations were found at the sampling sites 3 and 5. The observed highest distribution of polonium and uranium can be explained by the type of lichens thalli. The highest polonium and uranium concentrations were characterized for crustose thallus, the smaller for fruticose thallus, the smallest for foliose thallus. The results for polonium and uranium concentrations indicated great differences between analyzed species of organisms. Also, the polonium and uranium concentrations in all moss and lichen species were very diverse. The ability of accumulation of polonium and uranium isotopes by mosses and lichens makes them useful as bio-indicators of environmental radioactive contamination. ^210^Po is a product of the ^238^U decay series and is released into the atmosphere via the decay of ^222^Rn and these radionuclides on Sobieszewo Island are the result of human activity. The differences observed in ^210^Po and ^238^U concentrations in lichen and moss samples could be linked to the differences in accumulation properties of species and the sampling sites as it is shown as shows in the article there were significant differences in concentrations of ^210^Po and ^238^U among the sampling sites, kind of thalli as well as seasons and individual mosses and lichens characteristics.
